# Tripal v3: an ontology-based toolkit for construction of FAIR biological community databases

**DOI:** 10.1093/database/baz077

**Published:** 2019-07-22

**Authors:** Shawna Spoor, Chun-Huai Cheng, Lacey-Anne Sanderson, Bradford Condon, Abdullah Almsaeed, Ming Chen, Anthony Bretaudeau, Helena Rasche, Sook Jung, Dorrie Main, Kirstin Bett, Margaret Staton, Jill L Wegrzyn, F Alex Feltus, Stephen P Ficklin

**Affiliations:** 1 Department of Horticulture, Washington State University, Pullman, WA, USA; 2 Department of Plant Sciences, University of Saskatchewan, Saskatoon, SK, Canada; 3 Department of Entomology and Plant Pathology, University of Tennessee, Knoxville, TN, USA; 4 INRA, UMR IGEPP, BIPAA/GenOuest, INRIA/Irisa - Campus de Beaulieu, Rennes Cedex, France; 5 Bioinformatics Group, Department of Computer Science, University of Freiburg, Freiburg im Breisgau, Germany; 6 Department of Ecology and Evolutionary Biology, University of Connecticut, Storrs, CT, USA; 7 Computational Biology Core, Institute for Systems Genomics, University of Connecticut, Storrs, CT, USA; 8 Dept. of Genetics and Biochemistry, Clemson University, Clemson, USA

## Abstract

Community biological databases provide an important online resource for both public and private data, analysis tools and community engagement. These sites house genomic, transcriptomic, genetic, breeding and ancillary data for specific species, families or clades. Due to the complexity and increasing quantities of these data, construction of online resources is increasingly difficult especially with limited funding and access to technical expertise. Furthermore, online repositories are expected to promote FAIR data principles (findable, accessible, interoperable and reusable) that presents additional challenges. The open-source Tripal database toolkit seeks to mitigate these challenges by creating both the software and an interactive community of developers for construction of online community databases. Additionally, through coordinated, distributed co-development, Tripal sites encourage community-wide sustainability. Here, we report the release of Tripal version 3 that improves data accessibility and data sharing through systematic use of controlled vocabularies (CVs). Tripal uses the community-developed Chado database as a default data store, but now provides tools to support other data stores, while ensuring that CVs remain the central organizational structure for the data. A new site developer can use Tripal to develop a basic site with little to no programming, with the ability to integrate other data types using extension modules and the Tripal application programming interface. A thorough online User’s Guide and Developer’s Handbook are available at http://tripal.info, providing download, installation and step-by-step setup instructions.

## Introduction

Online repositories for biological data serve as a valuable resource for researchers as they provide a home for public data and offer services such as tools for data analysis, web services, or content for scientific community engagement. In 1988, the National Center for Biotechnology Information (NCBI) was created with the goal to ‘design, develop, implement and manage automated systems for the collection, storage, retrieval, analysis and dissemination of knowledge concerning human molecular biology, biochemistry and genetics’ (1). NCBI has served as a global repository for genomic sequence data, gene transcriptome data, genetic variance data and more for both human and non-human species for three decades.

Online community databases play a similar role but unlike large repositories such as NCBI, they serve a specific group of researchers focused on one species or a group of related species. In addition to housing data, these sites provide analyses, tools and outreach specific to their respective communities. In 1992, the FlyBase project developed one of the first community databases for genomic and genetic resources for *Drosophila melanogaster* (2, 3). Dendrome (now TreeGenes) initially begun in 1993 to house forest tree genetic maps and markers (4); the Rat Genome Database (RGD) (5, 6) in 1999 for rat research; The Arabidopsis Information Portal (TAIR), also in 1999, for genomic and genetic data for *Arabidopsis thaliana*, a model plant species (7). Later examples include the Saccharomyces Genome Database (SGD) in 2002 for the model species *Saccharomyces cerevisiae* (8); the Genome Database for Rosaceae (GDR) in 2003 for Rosaceae species including agriculturally important tree fruit and berry species (9); Knowpulse began in 2010 for pulse crop breeding (10); and many others.

In recognition of the commonalities among these community databases, the Genome Model Organism Database (GMOD) project was created in the early 2000s to help facilitate development of a common suite of software tools and infrastructure for model organism databases. GMOD provides outreach and training and is responsible for maintenance of Chado (11), an open-source relational database schema, originally developed for FlyBase. Chado provides a common data storage infrastructure, follows a normalized design and is meant to serve as a data warehouse for genomic, genetic and related biological and ancillary data. Several bioinformatics tools, especially those adopted by GMOD, are compatible with Chado and include InterMine, which integrates disparate biological data for construction of community data stores (12); GBrowse (13) and JBrowse (14) for whole genome visualization; and Apollo (15) for whole genome curation.

The advent of high-throughput sequencing technologies increased access to genomic, transcriptomic and genetic data and hence the desire for an increasing number of community databases for traditionally non-model species. However, three major difficulties confront non-model databases. First, budgets for non-model groups remain limited and do not support large development teams. Second, FAIR data principles (findable, accessible, interoperable and reusable) require metadata, cross-database integration and curation (16). For groups new to these principles, learning and implementing appropriate technologies and expertise can take years. Ontologies, or controlled vocabularies (CVs), serve a major role in FAIR data principles by supporting data integration, access and analysis. Unfortunately, use of ontologies to support data exchange may not be feasible for smaller groups due to limited curation time and tools or lack of expertise. Third, funding for long-term support of all organism databases has declined and sustainability for both model and non-model community databases is increasingly problematic. For instance, TAIR overcame funding challenges by offering a paid subscription model for sustainability (17). The model organism databases of FlyBase, the Mouse Genome Database (MGD) (18), SGD, The Gene Ontology Consortium (19), RGD, WormBase (20, 21) and the Zebrafish Information Network (ZFIN) (22) are unifying efforts under the Alliance of Genome Resources (https://www.alliancegenome.org/) to reduce duplication of effort among their member sites and to address challenges of sustainability. Similarly, the AgBioData group, a collection of more than 30 agricultural community databases work together to improve cooperation among members and recently published a set of recommendations (23). Without an improved sustainability plan, community databases may stagnate or disappear along with the valuable data they house.

Tripal (24, 25) is an open-source, freely available toolkit for the construction of online community databases and is meant to help address these challenges. Tripal is part of the GMOD family of tools and, by default, uses Chado as the primary data store. Tripal was first released in 2009 and was born from the need to create community databases for non-model species as high-throughput technologies supported greater access to genomic, transcriptomic and genetic data. The long-term goal was to provide a platform by which less funded, non-model research groups could create community databases of equivalent quality and resources to that of model species databases. Download, installation and setup instructions for Tripal can be found at the Tripal website: http://tripal.info.

Often community databases provide user portals by which researchers can log in, access data, submit content, communicate with one-another, access data via web services and interact with high-performance computing resources to analyze data. In order to support this level of interaction and to provide security standards, Tripal integrates with Drupal (http://drupal.org), a popular content management system (CMS). Drupal provides user authentication, security, content creation tools and an application programming interface (API) that allows a site developer to fully customize and create new content types. Tripal bridges Chado with Drupal and provides a variety of new tools and APIs for creation of community databases. This API allows a site developer to create their own extensions (or plugins) and share those with other Tripal users, thus reducing duplication of effort. By pooling resources towards the development of a common platform (i.e. Tripal) all stakeholder community databases improve the sustainability of each other.

With the adoption of Tripal by multiple overlapping communities, data federation using web services is increasingly important and useful for users and developers. For example, sites such as the GDR (9), TreeGenes (4), Hardwood Genomics Project (https://hardwoodgenomics.org/) and the Citrus Genome Database (26) house genomic, genetic and breeding data for their respective tree crop communities using Tripal. Similarly, Knowpulse (10), Cool Season Food Legume (27), PeanutBase (28) and the Legume Information System (29) house legume data using Tripal.

Data exchange and federation among online databases is challenging. Online databases each maintain their own data storage backend with unique integrity and referential constraints. Sites may refer to data entities (e.g. genes, germplasms, publications, etc.) and their properties (e.g. gene sequence, names, aliases, etc.) using different terminology and provide different hierarchical structures to represent relationships between entities and properties. Web services, which allow programmatic access to data in a site, may not exist and if they do exist, are typically incompatible for data exchange between sites. Therefore, sites that desire to exchange data must coordinate efforts in data storage and design of web services. This requires a substantial time investment for coordination. Tripal-based sites were not immune from these challenges. Despite a common data store back-end (i.e. Chado), not all sites housed the same data entities and especially, ancillary data (i.e. properties) for entities were not consistent. This is a natural side effect of the different data needs for different research communities and not a flaw with Chado or Tripal. For example, community databases with whole-genome assemblies and annotations store genes as data entities with properties such as genomic coordinates, functional annotations, cross-references, sequences and related publications. Sites without a whole genome may have genes but not necessarily genome coordinates or full sequences.

CVs and ontologies (CVs organized in hierarchies) offer an approach to improve and ease data discovery and therefore support data exchange. Chado was designed such that most data entities (e.g. genome features, stocks, genomic libraries, phenotypes, contacts, publications, etc.) are associated with a CV term that serves as a data ‘type’. Properties of a given entity are also associated with CV terms. For example, genomic feature entities are defined using the Sequence Ontology (SO) (30). Chado excels at storing genomic features and their ancillary data. It provides the database tables and documents recommendations for housing this type of data using the SO and other ontologies such as the Gene Ontology (GO).

Here, we report on the most recent version of Tripal (version 3). This version represents a major overhaul from previous versions, with the intent to aid site developers in adhering to FAIR data principles. CV-defined metadata and semantic relationships enhance the findability and accessibility of data stored in Tripal sites. This is further improved through discoverable web services and consistent data accessibility through both information pages and web services. Interoperability of data is facilitated through cross-site, heterogeneous data collections, consistent web services and shared CVs. Reusability is promoted by data loaders that require an existing analysis record prior to import intended for providing provenance details. Additionally, this version includes a new middle layer that allows interoperability between multiple storage methods facilitating storage of data that is not appropriate for relational databases such as Chado while ensuring a consistent user experience. The time it takes to setup a new Tripal v3 site will depend on the experience of the user, the quality of the input data and how well the data is standardized. Genomics-based sites are fastest to create as these data typically have standard formats, and Tripal is prepared to support them using existing data importers and a user-friendly web interface to organize them. Sites housing less standardized data may take longer to create as site developers may need to write data loaders or custom extensions. In summary, Tripal v3 is a powerful tool enabling non-model communities to create high-quality, FAIR compliant databases.

**Figure 1:**
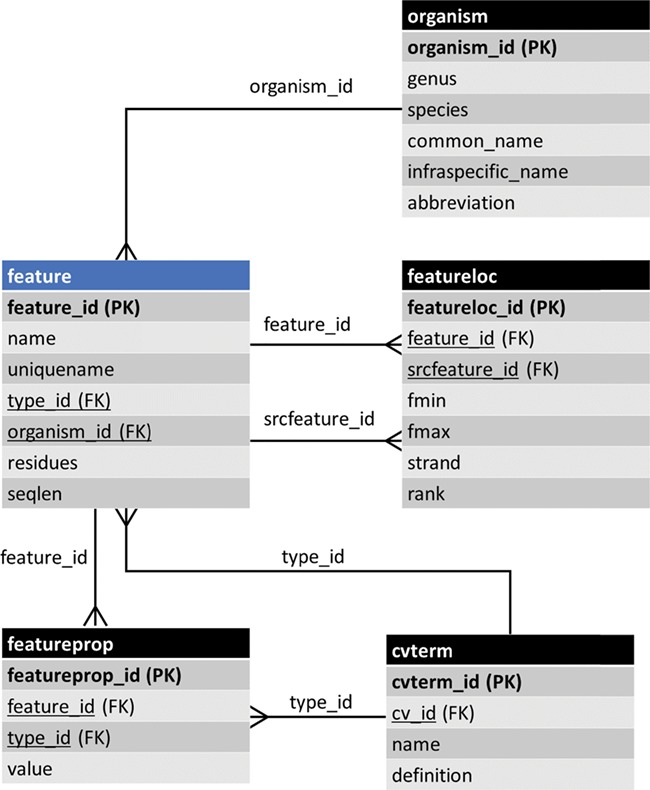
The entity–relationship (ER) diagram of the feature table of Chado and some of its linked tables (some table columns removed). The feature table stores genomic features and ancillary data is housed in linked tables.

**Figure 2:**
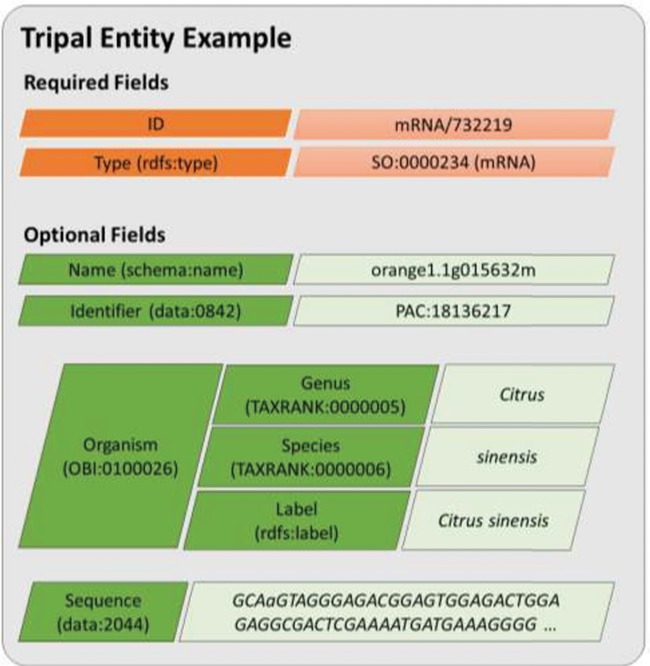
A diagram representing an entity model in Tripal v3. Each field consists of a key–value pair where the key must be defined using a controlled vocabulary term. The ID and type fields are required while all other are optional. Fields may have a single value or be a nested array of key–value pairs where keys must also use controlled vocabulary terms. This example includes real field data for the transcript named, orange1.1g015632m from the Citrus Genome Database.

## Implementation

### An ontology-based redesign

Data are accessible from a community database via two primary methods: first to researchers through a web page organized for viewing, and second to programmers via representational state transfer (REST) (i.e. RESTful) web services, where computer programs connect to the database, perform queries following a specific API and retrieve data. Prior to Tripal v3, data were presented to the user in a structure that was modeled after Chado tables. For example, Figure 1 shows the feature table of Chado and some ancillary-linked tables. The feature table is meant to house genomic features (e.g. genes, mRNA, Single Nucleotide Polymorphisms (SNPs), genetic markers, etc.) and has a set of linking tables for ancillary data such as the type of feature, the organism to which the feature belongs, properties for a feature, the genomic location (if applicable), etc. Prior to Tripal v3, a ‘feature’ page content type was responsible for displaying any record housed in the feature table of Chado. Thus, each gene page on a Tripal site was an instance of a feature page and a record in the feature table. While the researcher simply sees a ‘gene’ page, for instance, this approach is not conducive for data accessibility via computational means because it forces the consumer to understand data relationships using the constraints of a relational database model rather than a more natural semantic model. A semantic model is one in which the relationships among data uses CV terms in a subject-predicate format that links data together. It is more natural for humans and computers.

Rather than add support for semantic data exchange alongside the existing Chado-based human-designed page view, Tripal v3 was redesigned such that all data accessed either by a human or computer are provided via an underlying semantic-based data framework. This approach relies completely on CVs. This redesign models a distinct data object as an entity. For Tripal v3, an entity represents a single data object, such as a gene, germplasm, organism, publication, etc. By default, every entity requires two properties: a data type and a unique ID. The data type must be a term from an ontology or CV, and the ID is formed using the data type and a unique numeric ID. Thus, the ID, when combined with the site URL, ensures that every entity is uniquely identifiable on the world wide web. For example, the ID for the gene transcript (mRNA) named orange1.1g015632m at the Citrus Genome Database (and used as an example gene in the Tripal User’s Guide) is uniquely identifiable with mRNA/732219. Optionally, if more is known about an entity it may have any number of additional properties. The type of each property must be defined with a CV term as well. In Tripal v3, properties about an entity are referred to as fields. The value may be a single scalar (e.g. text or number), an array of scalar values, or an associative array of key/value pairs where each key is also a CV term and each value is any of the allowed value types. Figure 2 provides a visual model of a Tripal entity using data from the orange1.1g015632m transcript. Tripal v3 entities are grouped into content types of the same type, and the combination of a content type and its fields is referred to as a bundle. Thus, all gene pages or organism pages, for example, are bundles. In practice, the site administrator has complete control, using a graphical interface, over the number and types of optional fields that appear for each content type. This allows sites to customize pages to match the needs of their community without having to recode the various bundles and fields.

### Web services

RESTful web services are new to Tripal v3. A RESTful web service is one which uses the HTTP protocol (the same used by web browsers to deliver content) to query and retrieve data in a format that is usable by a computer program, most commonly, JavaScript Object Notation (JSON) and eXtensible Markup Language (XML) formats. Tripal entities and their fields use CV terms. This supports data accessibility and exchange by reducing ambiguity about the meaning of an entity. It also supports the concept of accessibility through Linked Data (31, 32), a concept used with the Semantic Web (33) to improve accessibility for data exchange. Linked data utilizes CVs and web URIs (universal resource identifier) to reference data objects (i.e. entities and field keys). By qualifying all data with URIs, the meaning of data can be resolved. For example, Figure 2 indicates that the example entity is of type SO:0000234, a term from the SO meaning: mRNA. A URL maintained by the SO exists for this term and further details can be found there. Likewise, each of the field keys are also defined using CV terms. For example, the name field uses the term ‘name’ from the Schema vocabulary (https://schema.org/). A lookup of the term ‘schema:name’ will resolve its meaning.

Tripal uses JSON-LD (34) (LD for linked data) to provide access to entities via its web services. JSON-LD is a data format for exchanging linked data using JSON that is a data-interchange format commonly used for RESTful web services. The following JSON provides an example for the orange1.1g015632m transcript. URLs have been shortened with an ellipsis for brevity (substitute ‘https://www.citrusgenomedb.org/web-services/content/v0.1’ in place of the ellipses):

{

"@context": ".../mrna.732219.json",

"@id": ".../mRNA/732219",

"@type": "mRNA",

"label": "orange1.1g015632m, PAC:18136217 (mRNA) *Citrus sinensis*",

"ItemPage": "https://www.citrusgenomedb.org/bio_data/732219",

"type": "mRNA",

"accession": "",

"organism": **{**

"label": "<i>*Citrus sinensis*</i>",

"genus": "Citrus",

"species": "sinensis"

**}**,

"name": "orange1.1g015632m",

"identifier": "PAC:18136217",

"sequence": ".../mRNA/732219/Sequence",

"sequence_length": "2075",

"sequence_checksum": "6c75d1779e249a7f102e75c32 18b39ad",

"is_analysis": **false**,

"is_obsolete": **false**,

"time_accessioned": "2011-03-22 09:22:42.725247",

"time_last_modified": "2011-03-22 09:22:42.725247",

"protein_sequence": ".../mRNA/732219/Protein_sequen ce",

"cds": ".../mRNA/732219/CDS",

"contact": **null**,

"database_cross_reference": ".../mRNA/732219/databa se_cross_reference",

"sequence_coordinates": ".../mRNA/732219/Sequence_ coordinates",

"location_on_map": **null**,

"annotation": ".../mRNA/732219/annotation",

"publication": **null**,

"relationship": ".../mRNA/732219/relationship",

"alternatename": **null**

}

As with all JSON returned by RESTful web services, data are organized by key–value pairs. However, to support linked data, this JSON contains three special keys: @context, @id and @type. The meaning of the @id and @type correspond directly with the ID and type of a Tripal entity. However, with linked data, each @id must be a URL. Here the @id is the unique URL for the entity on the site’s web services and uniquely defines this data object. The @context key contains a URL providing JSON that defines the linked data information. The following is a snippet of the JSON from that URL:

"mRNA":

"http://www.sequenceontology.org/browser/current_svn/term/SO:0000234",

"label": "rdfs:label",

"rdfs:label": "http://www.w3.org/2000/01/rdf-schema#label",

"ItemPage": "schema: ItemPage",

"schema: ItemPage": "https://schema.org/ItemPage",

"type": "rdfs:type",

"rdfs:type": "http://www.w3.org/2000/01/rdf-schema#type",

"data": "http://edamontology.org/data_",

"accession": "data:2091",

"data:2091": "http://edamontology.org/data_2091",

Using the JSON available via the @context link, the meaning of the keys used in the initial JSON is understood. For example, the meaning of the entity type "mRNA" can be found by following the provided link to the SO browser. The key "accession" resolves to the controlled vocabulary term "data:2091" that resolves to a URL for a term with the EDAM ontology. All keys in the JSON-LD data are found via the @context. Computer programs that support JSON-LD can use the context to reason about content.

In addition to JSON-LD, Tripal v3 web services use the World Wide Web Consortium (W3C) Hydra Core vocabulary (35) to make the web service discoverable. An active area of development for web services is the ability for a computer program to understand the data and functions available to it without prior knowledge of the web service API. A common bottleneck for data exchange is incompatible APIs such that a site developer who wishes to programmatically integrate data from multiple sources must learn the APIs of each source and write custom programs for each. A discoverable web services is fully traversable by a client program simply by providing the web service root. The Hydra core vocabulary was chosen for Tripal web services because it provides CV terms for collections (e.g. a listing of similar content types), the meaning of operations for an entity (e.g. HTTP POST, PUT, DELETE, etc.) and pagination. Any client that recognizes the Hydra vocabulary can traverse the web services of any Tripal site. The HydraConsole (https://www.markus-lanthaler.com/hydra/console/) is one such client that makes for quick browsing. Users of Tripal sites can learn to use the Tripal web services by following the instructions in the Tripal v3 User’s Guide, available on the http://tripal.info website.

By default, all entities are shared on a Tripal site’s web services via the content resource available by adding ‘/web-services/content/v0.1’ just after the base URL of the site. However, Tripal v3 provides a web services API that allows the site developer of a Tripal-based site to create new web services that are in turn discoverable. This API consists of a variety of PHP classes: TripalWebService, TripalWebServiceResource and TripalWebServiceCollection. Developers can create their own web services by implementing instances of these classes.

Tripal sites which enable Tripal web services follow FAIR data principles as data becomes ‘findable’ (i.e. discoverable) by any client program recognizing the Hydra core vocabulary. Furthermore, the JSON-LD linked data structure ensures accessibility by fully defining all metadata through CV terms. By redesigning Tripal with a semantic focus, data presented to the researcher on a typical information page matches what they would access programmatically. This further enhances accessibility since researchers can use the most suitable access model for their question.

### Storage API: Chado integration

**Figure 3:**
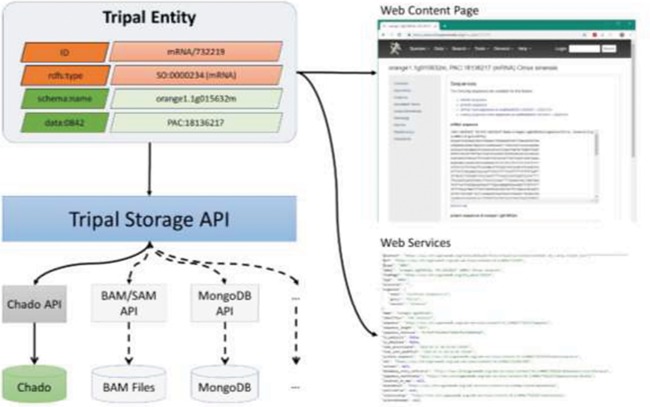
A diagram representing the Tripal Storage API and its relationship to content on a Tripal website. The Storage API sits between the Tripal Entity data model and the storage back-ends, allowing data to be integrated from multiple storage locations into a single entity.

Aside from the need to support data exchange, a long-term challenge for Tripal-based sites is increasing quantities of data, especially data not suitable for storage in traditional relational databases such as Chado. Examples of such data can include large-scale resequencing (as housed in BAM files), variant-data (as currently housed in VCF files) or large-scale network data that may be better suited for graph databases. As Tripal-based sites explore new avenues for data storage, Tripal needs to support those efforts. The new Tripal Storage API is intended to meet these needs. The Storage API allows Tripal to communicate with an underlying data store using a common set of PHP classes and functions. Figure 3 shows a diagram relating how the Storage API sits between the Tripal entity data model and the underlying data storage system. As shown in Figure 3, access to each database requires a database-specific API. Currently, only the Chado API has been written. However, other future APIs (represented via connection with a dashed line) can be developed and integrated into entities via the Storage API. The Tripal Developer’s Handbook, available at http://tripal.info, describes how developers can integrate new storage backends.

One of the most important components of the Tripal Storage API is the TripalField class. By default, Tripal provides a multitude of fields with each entity. These fields transmit the data stored in Chado for a given entity and are implemented using the TripalField, TripalFieldWidget and the TripalFieldFormatter classes. Almost all data housed in the Chado database can be displayed using these classes and Tripal provides user interfaces to allow a site developer to add new fields, hide unwanted fields and rearrange the display of fields on content type pages without the need for programming. However, sometimes a site developer may wish to add new fields that house data not supported by the current field classes. In this case, developers need only implement the three field classes. The instructions provided on the Tripal v3 Developer’s Handbook help developers implement their own fields. In addition, the Tripal Fields Generator package (36), available at https://github.com/tripal/fields_generator, will help automate initial setup of these field classes.

## Resources for site administrators

Tight integration of Tripal 3 entities and fields with Drupal provides a feature rich administrative user interface for managing the biological data pages. Through this interface, an administrator can choose which content types to make available on their site and customize the primary data storage location, as well as the CV term defining each type. Furthermore, for each content type the administrator chooses the ancillary data that is made available, its order and its format through a drag-and-drop interface. These administrative interfaces facilitate customization of Tripal-based sites to ensure they meet the specific needs of each community while reducing the amount of programming needed.

Documentation for Tripal site creation, management and customization is available through ReadtheDocs: https://tripal.readthedocs.io/en/latest/user_guide.html. In addition to thorough installation documentation, the documentation also demonstrates the flexibility and ease of customization provided by Tripal. Specifically, there is a tutorial-style guide demonstrating each step of setting up a genomics-focused site with documentation for breeding-focused sites in development. There are also guides for learning Chado, searching, materialized views, user file management, data loading and automated job execution. This documentation is continually expanding as new feature sets and community questions are voiced, ensuring that it is maintained and accurately reflects the current version of Tripal.

Using the Tripal API, a site developer can create reusable extension modules that expand the functionality of their Tripal sites and share those modules with the Tripal community. This sharing of extensions encourages co-development and reduces duplication of effort. A list of Tripal v3 compatible modules is listed in Table **1**. The authors did not create all of the modules listed in Table **1**. They may be published elsewhere and some may still be in development. An updated list of modules, as they become available is housed in the online Tripal documentation at https://tripal.readthedocs.io/en/latest/extensions.html. This listing helps serve as a gathering point for developers to collaborate on existing modules and as a resource for a site developer looking for additional functionality.

**Table 1:** Tripal v3 compatible extension modules, listed by category^*^

**Administrative**	
Tripal Alchemist	Transforms entities from one data type to another.
Tripal Curator	Splits properties and mass reassign property CV terms.
Tripal Headquarters	Supports admin approval of user submitted data.
**Functional annotation**	
Tripal Analysis Expression	Loads and visualizes NCBI Biomaterials and expression data.
Tripal Analysis Blast	Loads and displays XML results from the NCBI blast program.
Tripal Analysis KEGG	Loads and displays of KEGG ortholog assignments.
Tripal Analysis Interpro	Loads and displays of XML results from the InterProScan program.
Tripal CV-Xray	Maps content annotations onto browsable controlled vocabulary trees.
**Other data loaders**	
Genotype Loader	Loads genotype data housed in VCF files.
Mainlab Chado Loader	Provides various data loaders for Excel-based template files.
Raw Phenotypes	Loads phenotype data via Excel with validation, charts and downloads.
Tripal BibTeX	A BibTeX importer for publications.
Tripal Plant PopGen	Provides import of genotype, phenotype, environmental, etc. data.
Migrate Chado	Imports biological data to Drupal/Chado from other sources.
**Developer Tools**	
TripalDock	Creates a development Tripal site using Docker.
Tripal Download API	Provides an API for downloading Tripal/Chado data.
Tripal D3.js API	Provides d3.js integration for Tripal.
Tripal Fields Generator	Automates the generation of new Tripal fields.
Tripal Rapid Installer	Provides rapid installation of a Tripal site.
Tripal Test Suite	A framework for Unit Testing of Tripal modules.
**Third-party Integration**	
BrAPI	Implements the Breeding API for Tripal.
Tripal Blast	Provides a web interface for execution of BLAST.
Tripal Sequence Similarity	Provides a web interface for execution of BLAST and Diamond.
Tripal Galaxy	Integrates analytical workflows from Galaxy with Tripal.
Tripal JBrowse	Integrates JBrowse with Tripal.
VCF Filter	Provides custom filters for VCF files.
Tripal Apollo	Manages user accounts for your JBrowse Apollo instances.
Tripal Multi-Chado	Supports use of multiple Chado databases within a single Tripal site.
CartograTree	Provides a webapp to identify and visualize geo-referenced data.
**Searching**	
Mainlab Chado Search	Provides custom search forms for specific biological data.
Tripal ElasticSearch	Provides fast site-wide and cross-site searching of Tripal sites.
**Visualization/Display**	
Analayzed Phenotypes	Visualization for large scale phenotypic data.
TripalMap	Visualization of genetic maps.
CvitEmbed	Integrates CViTjs to provide whole-genome visualizations.
Mainlab Data Display	Provides custom displays for many biological data types.
ND Genotypes	Visualization of genotypic data.
Tripal Fancy Fields	Provides charts (e.g. pie, donut, or bar chart) and tables for data.

^*^Listed modules are not necessarily created by the authors of this manuscript and may be published elsewhere. Please see https://tripal.readthedocs.io/en/latest/extensions.html for an updated listing and links to source code repositories.

Regarding data exchange, here we provide some additional details for the ElasticSearch extension module. Tripal v3 was developed to encourage data accessibility and sharing and the ElasticSearch module enhances this ability. ElasticSearch (37) is a free, open-source, highly customizable RESTful search engine. The Tripal ElasticSearch module (38) handles much of the customization necessary to enable indexing of Tripal entities. This module makes it easy for a site administrator to connect an ElasticSearch instance to their site and manage content indexing. In addition, Tripal ElasticSearch provides cross-site data exchange. Administrators need only opt-in to sharing their data to expose site indexes to other Tripal v3 sites to allow cross-site search on their own site. This greatly enhances interoperability between data and data re-use thus facilitating the FAIR data principles.

## Resources for site developers

Tripal extends the Drupal API to facilitate customization and extension of Tripal sites. For example, developers can use the API to create custom data storage solutions, ancillary data fields and visualizations, access data in Chado, develop custom data importers and generally customize every aspect of a Tripal site. The API is well documented with dedicated pages for each function and class (http://api.tripal.info/api/tripal/3.x), as well as tutorial-style developer examples (https://tripal.readthedocs.io/en/latest/dev_guide.html). Additionally, best practices have been established to help developers create extension modules that can be shared with the community and contribute to the core Tripal package (https://tripal.readthedocs.io/en/latest/dev_guide/contributing/pull_requests.html). Community discussions in the GitHub issue queue (https://github.com/tripal/tripal/issues), Tripal Help Desk calls and monthly Tripal Community meetings provide an inclusive environment for collaboration.

To ensure robustness of code, PHPUnit has become the testing framework of choice for Drupal. However, implementing it in Drupal 7 has been historically difficult due to the lack of core infrastructure. Tripal Test Suite (36) bootstraps a Tripal site to use PHPUnit and lessen the development time needed to add PHPUnit tests to any Tripal extension module. Tripal v3 core package uses Tripal Test Suite to provide core tests. Furthermore, Tripal Test Suite also facilitates the development of unit tests for Tripal extension modules and provides conveniences like name spacing, database seeders, transactions and data factories. This framework allows developers to confirm that their extensions work as desired and to maintain quality.

To ease development and deployment, a set of docker images were developed (https://github.com/galaxy-genome-annotation/docker-tripal). These images are based on an up-to-date PHP image and the latest Drupal 7 releases. The image is compatible with the other docker images built in the frame of the Galaxy Genome Annotation project (https://galaxy-genome-annotation.github.io/). The set of images can be instantiated using docker compose and include a service for Tripal and Drupal, another for PostgreSQL and Chado and a third containing ElasticSearch. Tripal docker is designed for production usage and is configured for advanced cache management to improve performances. It is used on various public resources including the insect genomic resources provided by the BIPAA platform (https://bipaa.genouest.org).

While Tripal and Drupal are PHP applications, the Python-tripal package (https://github.com/galaxy-genome-annotation/python-tripal) provides access to Tripal via a Python interface. It provides command-line interface (CLI) access to Tripal using the web services provided by Tripal and the tripal_rest_api module (https://github.com/abretaud/tripal_rest_api). Python-tripal can import various data (e.g. annotations in GFF format, sequences in FASTA format, orthology or expression data) into a remote Tripal server. It also allows users to explore the content of a Tripal instance programmatically (e.g. entities export). The Python-tripal tool is used with the Galaxy Tools package (https://github.com/galaxy-genome-annotation/galaxy-tools/tree/master/tools/tripal). The Python-tripal library can be used to load data from Galaxy into a remote Tripal server.

## Challenges

Creation of an online genome database is relatively straightforward with Tripal v3 as step-by-step instructions are provided in the online User’s Handbook, data file formats are well defined (e.g. GFF, FASTA, etc.) and ontologies are well defined (e.g. SO and GO). Tripal provides out-of-the-box loaders for genomic data, and best practices for storing genomic data in Chado are defined. Moreover, Tripal 3 provides an administrative interface that allows the site administrator to organize content pages as desired, and Drupal provides a variety of themes to change the look-and-feel of the site.

Often, however, community databases desire to support more than just genomic data. Chado provides a variety of database tables to support these data including tables for expression data, stocks, germplasm, genotypes, phenotypes, analyses, projects and more. Tripal v3 supports all Chado tables and supports custom tables as well. Yet, biological data is complex and organizing that data in Chado can be challenging. For example, the natural diversity tables of Chado were added to version 1.2 to associate phenotype and genotype data for natural diversity studies (39), yet its adoption has been challenging due to the size and scale of data. New users of Tripal who expect to move beyond genomic data will need to learn the structure and expectations of the Chado tables. In some cases, best practices for storage of some data types are not well defined. Both the Tripal and Chado communities actively discuss these best practices and new site developers are encouraged to participate.

In some cases, a site developer may want to create new tools, visualizations or extensions for their Tripal sites to support custom community needs. Tripal provides an API that gives access to the underlying data and to the various tools that Tripal provides. Drupal also provides an API that developers will need to learn. Often, Tripal-sites are housed in academic institutions where principal investigators have access to undergraduate computer science students or bioinformaticists with primarily data analytics experience. Both types of individuals may have difficulties learning Drupal. Therefore, a site developer should have programming experience prior to learning Drupal as the Drupal API is complex, and the training period may take several weeks.

Ontologies and CVs are critical to make data findable and interoperable. However, vocabularies are dynamic (they change), they sometimes do not have all terms needed, and some terms are in multiple ontologies. When a term is missing, it is recommended that users communicate with developers of the vocabulary to add needed terms. But in the meantime, Tripal does support use of locally created vocabularies, and each site may create temporary local terms to share their data. However, these challenges limit the ability of sites to recognize shared data. Unfortunately, this is the problem all sites confront—including non-Tripal sites. For inter-site data exchange, site developers may need to communicate with each other to ensure compatibility of terms. However, the entity and field organization of data allows Tripal sites to recognize data when terms are in common and provide default views when terms are not.

Finally, Drupal is a powerful open-source CMS and is quite large. In order to stay at the forefront of new technology, Drupal is not backward compatible with previous versions. This has required that a site developer rewrite portions of their extension modules as major version releases occur. This has proved challenging when Drupal upgraded from version 6 to 7 and then from 7 to 8. Drupal core developers have indicated, however, that the change from 8 to 9 and future updates are expected to be more seamless.

Despite these challenges, over 140 sites currently report using Tripal for either development or production for a variety of community databases. Sites whose developers actively participate in Tripal community events house data for over 4300 species from a diverse range of plants, fungi and animals. Often these developers collaborate to provide help for other developers. The Tripal community meets monthly, provides online training and help desk sessions, offers discussion forums for best practices and has yearly face-to-face coding and planning meetings that are open to all. A site developer who chooses to become involved can draw on this collective experience, and in turn contribute back to the larger community, contributing to the sustainability of all Tripal sites.

## Conclusion

Tripal is a powerful tool for non-model organism communities. Through Tripal, communities receive many features aiding in the creation of high-quality, FAIR-compliant biological databases. The semantic focus of Tripal 3 enhances the quality of data sets by ensuring data and metadata are fully described using controlled vocabulary terms. Discoverable web services, cross-site data collections and consistent access of data across storage models make disparate data both interoperable and findable. Furthermore, Tripal addresses the sustainability concerns many biological databases face through shared development input and support of an open-source, community-developed maintenance model. Tripal continues to respond to the needs of community biological databases with a community-wide discussion ongoing for Tripal version 4.
